# Analyses of crop water use and environmental performance of small private irrigation along the white Volta basin of Northern Ghana

**DOI:** 10.1016/j.heliyon.2023.e19181

**Published:** 2023-08-16

**Authors:** Abdul-Rauf Malimanga Alhassan, Andrew Manoba Limantol, Isaac Larbi, Rosemary Anderson Akolaa, Gilbert Ayine Akolgo

**Affiliations:** a University of Environment and Sustainable Development, Department of Water Resources and Aquaculture Management, Somanya, Ghana; b University of Environment and Sustainable Development, Department of General Studies, Somanya, Ghana; c University of Energy and Natural Resources, Department of Agricultural and Bioresources Engineering, Sunyani, Ghana

**Keywords:** Informal irrigation, Government irrigation, Water saving, Crop productivity, Environment, Sustainability

## Abstract

Small private irrigation (SPI) is a farmer-initiated irrigation which has the potential to increase the contribution of the overall irrigation sector to global food security. However, there is no much information about these systems for effective policies for regulation. This study compared the resource use productivities and environmental impacts of SPI systems to those of a government-led irrigation scheme (GIS) in Northern Ghana. The results showed that land productivity was higher in the SPI than in the GIS. Productivity per unit cultivated area was 2571.00 US$/ha under SPI while that of the GIS was 676.00 US$/ha. Output per unit command area was also two times higher in the SPI than in the GIS; that is 2571.00 US$/ha and 1113.00 US$/ha for SPI and GIS respectively. For water productivity, output per unit irrigation supply was 0.33 US$/m^3^ and 0.08US$/m^3^ for SPI and GIS respectively while output per unit water consumed by ET was 0.60 US$/m^3^ for SPI and 0.06 US$/m^3^ for the GIS. The results implied that the SPI schemes performed better in land and water productivities compared with the GIS which is attributed to higher yields and the selection of high valued crops by farmers under SPI. However, both irrigation system types at the time of this study did not cause significant deterioration to the water bodies and surrounding environment as the biochemical oxygen demand (BOD) values of nearby water bodies were less than 3.0–5.0 mg/l, which is considered as acceptable levels for drinking water by World Health Organisation (WHO) while salinity levels were also within acceptable limits (<750 μS/cm). With appropriate policies to regulate and provide support systems to the SPI, these systems may increase the overall agricultural productivity and improve job creation for the teeming unemployed youth and women in the savannah agroecological zone of Ghana.

## 1 Introduction

Irrigation plays a central role as a source of food and fiber for the peoples of the globe. The world gets 40% of its food supply from irrigation even though it covers only 20% of the world's cultivated area [1]. In the 1970s, so much public funds were invested in the construction of large public irrigation schemes, but this trend eventually slowed down due to poor performances of these big systems [1]. The world demand for food however continued to rise. It is estimated that, by 2050 food supply must increase by 70% in order to meet the surging demand [1]. Irrigated agriculture is still expected to be an important source of food and fiber. While the formal irrigation sector is a major strategy in this course, the contribution of the informal irrigation sector cannot be overemphasized. In India, private farmers have developed millions of wells for private irrigation over the past 40 years [2]. The trend is similar in Sub-Saharan Africa where small private irrigation (SPI) is gaining popularity amongst local farmers in rural and urban dwellings. An estimated 5 million small scale farmers in the sub-continent use low technologies to cultivate 1 million ha of land under irrigation [3]. The sector is therefore expanding quickly but without much attention and regulation [2]. De Fraiture et al. [4] intimated that, SPI is taking centre stage in irrigation development around the globe yet it has received little recognition. Policy makers, donor community and researchers have not yet turned their attention towards the sector, leading to little understanding of the systems including the impacts, challenges, risks, equity issues, efficiencies and environmental consequences [3]. It has been reported that perception of negative environmental impacts related to irrigation farming is a significant deterrent for adoption of irrigation [5]. Also, if the benefits of an irrigation system are not too clear, it can also influence adoption negatively. It is thus imperative to conduct performance appraisals for better understanding of these systems.

Performance assessment is a widely used concept in the management of irrigation and drainage systems. The concept first evolved in the industrial sector where performance-oriented processes were targeted at accomplishing process functions with less resources and time [6]. The concept was applied to the irrigation sector giving birth to a number of frameworks. Bos et al. [7] defined their concept of assessment of irrigation and drainage systems as " the systematic observation, documentation and interpretation of the management of an irrigation and drainage system, with the objective of ensuring that the input resources, operational schedules, intended outputs and required actions proceed as planned”. They wet further to develop a general framework for a diagnostic” type of irrigation performance assessment where they indicated that the purpose of the assessment and the strategy to use must be clearly stated before any assessment takes effect. Their concept is anchored on some background questions such as " the purpose”, “for whom it is being carried out”, “whose view point is used”, “who is going to carry out the assessment”, “the type of assessment” and the “extent of the assessment”. Small and Svendsen [8] categorised performance assessment of irrigation and drainage systems under four types. These include: accountability, operational, intervention, and sustainability. These are applied in a number of ways and settings and they also depend on your purpose and objective of the assessment. One example of the applications of performance assessment is the comparative assessment of one, two or more irrigation systems with another in order to set suitable bench mark standards or to undertake a diagnostic process of what is being done right at one system but not the other. Appropriate indicators will have to be selected after identifying the purpose of the assessment. The application of performance assessment indicators depends on the type of assessment intended. In line with this [9], developed nine indicators comprising of four external and five other indicators for comparative analysis of irrigation systems. Malano and Burton [10] also outlined a set of 23 indicators for benchmarking performance of irrigation and drainage systems. A rapid appraisal procedure (RAP) was also developed by Burt et al. [11]. A more comprehensive process known as benchmarking of technical indicators (BMTI) of irrigation systems was also developed by Gonzalez et al. [12] which combines RAP, benchmarking guidelines and report card process for feedback analysis. However, in this study we employed the indicators developed by IWMI which seems most suitable for comparative studies due to its simplification and standardization of the indicators. The introduction of the standardized gross value of production (SGVP) ensures cross-comparison of irrigation systems, both locally and internationally. The SGVP also allows for future comparisons.

This study thus conducted a comparative performance analysis of land and water productivities of small private irrigation (SPI) systems along the White Volta basin and the Bontanga government irrigation scheme (GIS) of Northern Region of Ghana. The results of this study will ensure a better understanding of the contributions and implications of the system type on productivity and the environment for better regulation and policy direction.

## 2 Materials and methods

### 2.1 Study area description

**Fig. 1:**
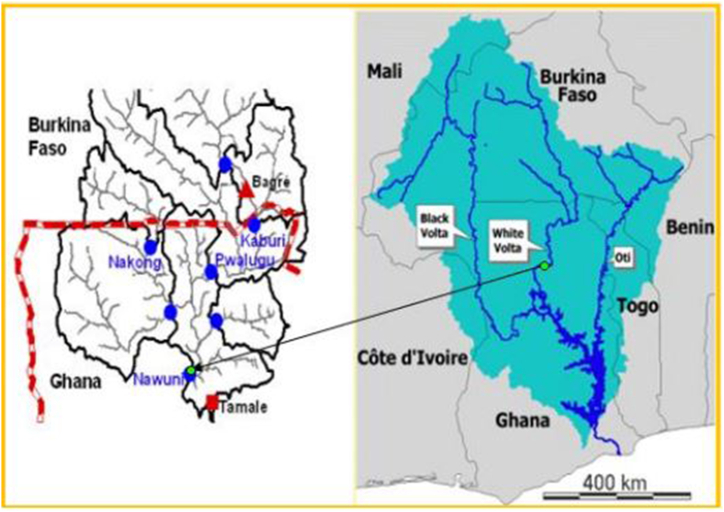
Location of nawuni catchment (left) within the volta river basin (Right) Modified from Ref. [18] (left) & [19] (right).

**Fig. 2:**
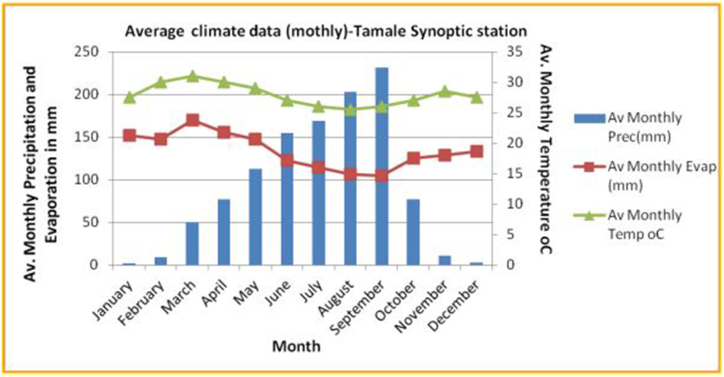
Climatic characteristics of the Tamale synoptic station (Climatic data was taken from FAO_NewLocClim).

This study was conducted in the Nawuni catchment of the transboundary White Volta Basin (WVB) in the Ghanaian part of the basin (Fig. 1). The catchment is located between Latitude 9° 87ʹ N to 11° 15ʹ N and Longitude 0° 5ʹ W to 1° 26ʹ W [13] with a surface area of 96,230 km^2^ [14]. The Nawuni catchment is the largest of all the catchments within the WVB and is characterized by a fairly low relief with moderate elevation in few parts of the north and east of the catchment given it a mean elevation of about 200 m [15]. Like other parts of the WVB, the climate of Nawuni catchment is driven the Intertropical Convergence Zone (ITCZ) air mass that controls the climate of the West African region [16]. As shown in Fig. 2, the Nawuni catchment is characterized by unimodal rainfall pattern that occurs between April–October and peaks in August/September with a long dry period between November and March. The mean annual rainfall in the area is approximately 978.83 mm [17] with about 80% of it occurring between June and September [16]. The mean daily temperature in the catchment is between 26 °C and 32 °C, and average annual potential evapotranspiration of 1800 mm with monthly amounts exceeding rainfall in nine months [16]. The dominant soils of Nawuni catchment are generally good for agriculture which is the main occupation of the inhabitants [15].

The Nawuni catchment hosts several small-scale private dry season irrigation farmers who draw water from the catchment with motor pumps. Most of these farmers are vegetable producers and engage in dry season vegetable cropping along the river banks, usually between October and March. The dominant vegetable crops cultivated are okra, pepper and onions. Pumping machines and poly vinyl chloride (PVC) pipes are the means of water supply from the river to the farms for irrigation. For the purpose of this study, the activities of these farmers are termed as “small private irrigation” (SPI).

The Bontanga Irrigation Scheme is a government-led irrigation scheme (GIS), also located in the catchment, specifically between latitude 9° 30′ and 9° 35′N and longitude 1° 20′ and 1° 04′W. It is one of the 22 government schemes under the management of the Ghana Irrigation Development Authority. The scheme has a reservoir with an estimated maximum storage capacity of 25 million m^3^ with an outlet structure consisting of 2 main canals with length of about 6 km each and 28 laterals or secondary canals [20]. It has an irrigable command area and irrigated cropped area of about 450 ha and 390 ha respectively [21], and hosts farmers from 17 communities, most of whom cultivate continually all year round. Farmers under the scheme mainly cultivate paddy rice during the dry season and vegetables such as pepper, okra, onion, and tomato are included during the wet season.

## 3 Data collection

### 3.1 Cultivated area, production data and crop prices

Cultivated areas, average yield and crop prices were needed to determine agricultural productivity. Production data were gathered through a combination of structured questionnaires, focus group discussions and field measurements. A Global Positioning System (GPS) was used to measure on-site cultivated areas for 2013 under the SPI whilst cultivated areas under the GIS were obtained from records from the project management. Crop prices were obtained through focus group discussions as well as from secondary sources. Three-year average yield (2011–2013) was obtained through farmer interviews by the use of structured questionnaires. For the SPI, purposive sampling was done and three of the five sites (Dipale, Kuli and Walshei sites) were selected for the interviews, representing Savelugu, Kumbungu and Tolon districts respectively within the catchment. A total of 45 households were selected for interview from the three sites. For the GIS, 50 farmers were interviewed. Farmers were selected by obtaining a list of cultivators from the agricultural extension officer and the required number of farmers were selected through random sampling. Crop yields were estimated by obtaining information on the quantity of produce harvested within the past three seasons in local units such as bags and buckets which were converted to metric units i.e. in kg/ha. Three-year (2011–2013) farm-gate market prices were taken from statistics, research and information department (SRID) of the ministry of food and agriculture (MoFA) for rice, pepper, onion, tomato and okra. Farm gate prices of crops were also solicited from farmers through focus group discussions for validation purposes.

### 3.2 Estimation of crop water requirement

Crop water requirement (CWR) was calculated for the various crops using FAO cropwat 8.0 computer-based model. The model uses climatic, crop and soil data to estimate the potential evapotranspiration. The model uses the FAO Penman Montheith method to estimate the reference evapotranspiration (ETo) which was multiplied by the crop coefficients (Kc) to get the Potential Evapotranspiration (PET). To run the cropwat 8.0 model, long term climatic data were downloaded from the FAO software New_LocClim which included monthly rainfall amounts (mm), average minimum and maximum temperature (^o^c) on monthly basis, average monthly humidity in %, average monthly wind speed (km/day) and monthly average sunshine hours (hrs). The Tamale synoptic station was the nearest to the study area, thus data from the station was used. It is located at latitude 9.50 N and longitude 0.85 W. The rainfall data was also used to estimate the effective rainfall using the United States Department of Agriculture Soil Conservation (USDA-SC) method. Crop data such as crop type, planting dates, rooting depth (m) and crop development stages (days) were also used as input data in Cropwat model. These were gathered by conducting farmer interviews and field observations. Soil moisture at field capacity, permanent wilting point and saturation (mm/m) are also inputs required for CWR calculation and were gathered from Savannah Agricultural Research Institute (SARI) for the Bontanga irrigation scheme and Kukobilla sites along the White Volta River. The predominant soil type, maximum rain infiltration rate (mm/day), and maximum rooting depth of the crops were generated from default data in the cropwat 8.0 model.

### 3.3 Annual total volume of water diverted for irrigation

Water use data for the SPI was gathered by checking the flow rates (m^3^/h) of farmers' pumps, number of hours of irrigation per day and the number of times of irrigation per week. The average suction plus delivery head was also observed while losses were also calculated. These were used together with pump characteristics from manufacturer's manual to estimate the total amount of water pumped out of the river per season. For the GIS, water use data was gathered by discharge measurements on-site using the Velocity-Area method with a floating object with a correction factor of 0.8. The discharges measured were then used to estimate the total amount of water diverted for irrigation in both systems.

### 3.4 Selected comparative performance indicators

A number of external indicators as described by Ref. [9] were used for the assessment as shown in equations (1), (2), (3), (4), (5), (6)). The indicators were selected due to the ability to make comparison with such indicators. Furthermore, the data required for calculation of those indicators were easily accessible. This makes these indicators cost-effective and less time consuming in assessment. The indicators are as summarised below.

### 3.5 Land productivity

In order to ascertain the outputs relative to the land cultivated and the land available to the farmers, the output per unit cropped area which is the cumulative gross value of production in relation to the total land cultivated was computed using equation (1) whiles equation (2) was employed in computing output per unit command area, which is the cumulative gross value of production in relation to the total land available to the farmers. The cumulative gross value of production is as shown in equation (3).

(1) $$Output\;per\;cropped\;area=\frac{SGVP}{Cropped\;Area}\;\left(\frac{\$}{ha}\right)$$

(2) $$Output\;per\;unit\;command\;area=\frac{SGVP}{Command\;area}\;\left(\frac{\$}{ha}\right)$$

(3) $$SGVP=\sum_{crops}\left[A_{i}x\;Y_{i}x\;\frac{P_{i}}{P_{b}}\right]x\;P_{world}$$

where SGVP = Gross Value of production standardized to world market value ($).

A_i_ = Area cultivated under crop i (ha), Y_i_ = Yield of crop i (kg/ha), P_i_ = Local Price of Crop i in Ghana cedis (GH'), P_b_ = Local price of the base crop (GH'), P_world_ = World market price of the base crop ($), Cropped Area = Total area cultivated under all crops per season (ha), Command area = Total area cultivated under all crops per year (ha).

### 3.6 Water productivity

The outputs per unit water used were computed using output per unit irrigation supply (equation (4)), which indicates the cumulative gross value of production relative to the total amount of water diverted for irrigating the farms, and output per unit water consumed (equation (5)) which is the cumulative gross value of production relative to the actual water consumed by the plants.

(4) $$Output\;per\;unit\;irrigation\;supply=\frac{SGVP}{Diverted\;Irrigation\;supply,V_{\mathrm{div}}}\;\left(\frac{\$}{m^{3}}\right)$$

(5) $$Output\;per\;unit\;water\;consumed=\frac{SGVP}{Volume\;of\;water\;consumed\;by\;plants}\left(\frac{\$}{m^{3}}\right)$$

### 3.7 Environmental performance

The environmental performance was based on water quality parameters such as salinity levels of the irrigation water which is measured by the electrical conductivity of the water (ECi) and Biochemical Oxygen Demand (BOD). The salinity levels were used to calculate the rate of salt accumulation into the soil due to the source of irrigation water. Microsoft Excel was used to calculate the average BOD values and the difference between BOD of upstream and downstream sections of White Volta. The accumulated salt due to irrigation was estimated by the relation in equation (6) [22].

(6) $$Accummulated\;Salt\;due\;to\;irrigation=V_{\mathrm{div}/ha}x{EC}_{i}x\;6.40\;x\;10^{-4}\;\left(ton/ha/year\right)$$

where.

EC_i_ = Electrical conductivity of the irrigation water in ds/m.

V_div/ha_ = Volume of water diverted for irrigation in m^3^ per hectare per year.

## 4 Results

### 4.1 Average yield, crop prices and cropping pattern

Three-year average yield of okra, pepper, onion, tomato and rice were 4515.1 ± 623.6 kg/ha, 3487.9 ± 189.3 kg/ha, 4471.3 ± 1419.7 kg/ha, 3505.4 ± 160.6 kg/ha and 2916.9 ± 404.2 kg/ha respectively under GIS whilst under SPI average yields were 4204.4 ± 301.5 kg/ha, 4522.1 ± 72.2 kg/ha and 3615.3 ± 146.5 kg/ha respectively for okra, pepper and onion (Table 1). Among the common crops grown in both systems, average yields of okra and onion were not statistically significantly different but average yield of pepper of the SPI was significantly greater than the average yield of pepper of the GIS at *p* < 0.05 (Table 2).

**Table 1:** Three-year average yield (2011–2013) of main crops under GIS and SPI.

Irrigation System	Average Yield (kg/ha)				
	Okra	Pepper	Onion	Rice	Tomato
**GIS**	4515.1 ± 623.6	3487.9 ± 189.3	4471.3 ± 1419.7	2916.9 ± 404.2	3505.4 ± 160.6
**SPI**	4204.4 ± 301.5	4522.6 ± 72.2	3615.3 ± 146.5	NA	NA

SPI means Small Private Irrigation GIS means Government Irrigation Scheme.

**Table 2:** T-test analysis for crop yields in GIS and SPI.

Crop	t-statistic	t-critical (two-tail)
Okra	−0.762	2.005
Pepper	2.619	2.004
Onion	−2.273	2.015

Average prices of the crops showed that pepper had the highest price compared with the other crops. Average crop prices were 1.60, 3.80, 2.20, 2.40 and 0.60 GH'/kg for okra, pepper, onion, tomato and paddy rice respectively across sites (Table 3). Pepper had the highest monetary value per kg while paddy rice had the least value.

**Table 3:** Average crop price (2011–2013) in the study area.

Crop	Average price (GH'/kg)
Rice	0.60
Okra	1.60
Pepper	3.80
Onion	2.20
Tomato	2.40

The cropping pattern showed that farmers in the GIS cultivated only paddy rice, that is 100% of the land was used for rice cultivation in the wet season. In the dry season, 74% of the total cultivated area was put under rice cultivation, while 26% was used for cultivating other crops including okra, pepper, onion and tomato. Pepper was the least cultivated covering only 2.5% of the total cultivated area. In the SPI, crops grown included mainly vegetables with 31% of the total area under Pepper, 54% under okra, while the remaining 15% was used for onion and tomato production.

### 4.2 Total water supply under GIS and SPI

Table 4 shows the total water used in both the GIS and SPI. An estimated total irrigation amount of 7.73 Mm^3^ of water was diverted annually for irrigation in the GIS while total rainfall utilization both in the dry and wet seasons amounted to 4.00 Mm^3^ which resulted in a total water used in the scheme of 11.73 Mm^3^. In the SPI, the total water abstracted from the river for the 65 ha is 0.5 Mm^3^ for the dry season. Total rainfall contribution in the dry season is 0.09 Mm^3^ which gives total amount of water diverted from the White Volta River for small private irrigation of 0.59 Mm^3^.

**Table 4:** Total water supply under GIS and SPI.

Irrigation Scheme	Total Area cultivated (Ha)	Total rainfall (Mm^3^/year)	Total Irrigation supply (Mm^3^/year)	Total water supply (Mm^3^/year)
GIS	938.4	4.0	7.73	11.73
SPI	65.0	0.09	0.50	0.59

### 4.3 Land productivity

**Fig. 3:**
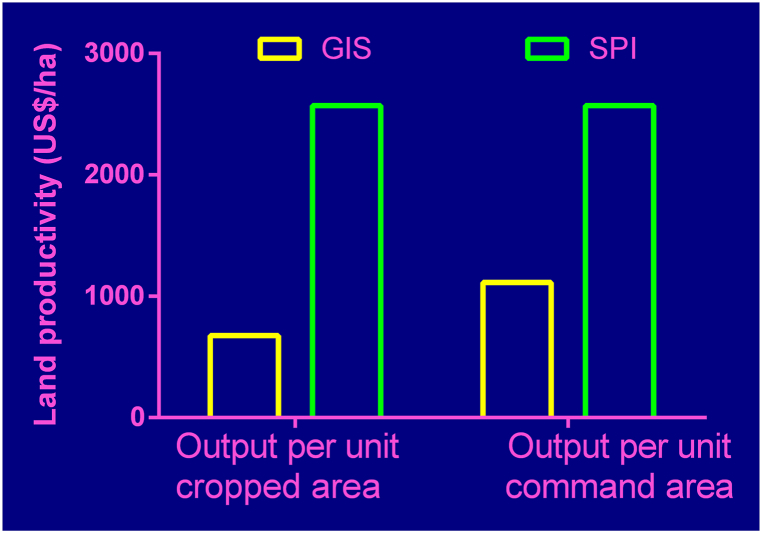
Output per Unit Cropped Area and Output per unit Command Area GIS means Government Irrigation Scheme SPI means Small Private Irrigation.

Two main indicators were used for comparison of overall output of land under the government scheme and under small private irrigation. The indicators include output per unit cropped area and output per unit command area. The SGVP was used to assess the overall scheme/site output. It is the sum total of the values of all products harvested in the scheme/site expressed in monetary terms (US$) with reference to a local crop tradable in the international market. The results indicated that the standard Gross value of Production (SGVP) of US$ 634,297.90 was achieved by cultivating an area of 938 ha (ha) of land in the GIS while under SPI, US$ 167,083.64 from 65 ha of land was achieved (Table 5). The results of SGVP for the systems are shown in Table 5. Output per unit cropped area for the GIS was 676.00 US$/ha while average output per unit cropped area for SPI was 2571.00 US$/ha (Fig. 3). While output per unit command area for the GIS increased to 1113.00 US$/ha, that of the SPI remained as 2571.00 US$/ha as shown in Fig. 3.

**Table 5:** Standard gross value of production under GIS and SPI.

Crop	SGVP (US$)	
	GIS	SPI
Rice	364,531.90	
Okra	96,456.44	59,869.09
Pepper	80,641.53	87,054.55
Onion	63,075.60	20,160.00
Tomato	29,592.44	
**Total**	**634,297.90**	**167,083.64**

#### 4.3.1 Water productivity

**Fig. 4:**
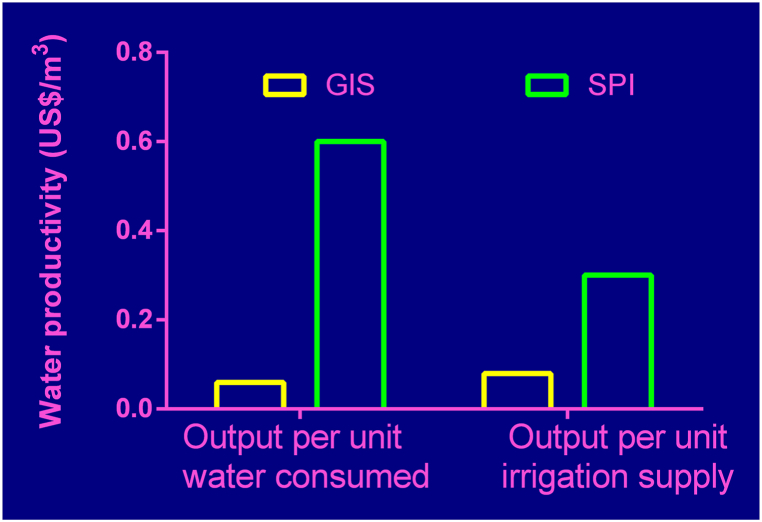
Output per unit irrigation supply and output per unit water consumed by ET.

Water productivity was analyzed using two indicators (1) Output per unit irrigation water supplied and (2) Output per unit water consumed by plants to meet crop water requirement. The total water used and the irrigation applied in both systems are compared to the outputs generated in United States dollars. The total quantity of water used in the systems amounted to 11.70 million cubic meters (Mm^3^) in GIS for two seasons and 0.59 Mm^3^ in the small private irrigation sites for only the dry season. The GIS achieved an output per unit irrigation supply and output per unit water consumed of 0.08 US$/m^3^ and 0.06 US$/m^3^ respectively. The SPI achieved averagely higher output compared to GIS. Output per unit irrigation supply was 0.33 US$/m^3^ while as output per unit water consumed was 0.60 US$/m^3^ as shown in Fig. 4.

#### 4.3.2 Environmental performance

Electrical Conductivity (EC) and Biological or Biochemical Oxygen Demand (BOD) of irrigation water can be used as parameters for assessing environmental performance of irrigation systems. The EC of irrigation water of the Bontanga Government Irrigation scheme was 63.1 μS per centimetre (μS/cm) while three sites along the White Volta where small-private irrigation is practiced ranged between 54 and 87 μS/cm and these resulted in salt accumulation of 0.33 ton/ha annually in the government scheme while that of the small private irrigation sites ranged between 0.27 ton/ha and 0.43 ton/ha annually (Table 6).

**Table 6:** Electrical conductivities and salt accumulation at GIS and SPI sites.

Scheme	EC (μs/cm)	Volume of water diverted for irrigation (m3/ha)	Salt Accumulation (ton/ha/y)
Bontanga (GIS)	63.10	8239.00	0.33
Kukobilla (SPI)	54.20	7680.00	0.27
Dipale (SPI)	73.60	7680.00	0.36
Walshei (SPI)	87.20	7680.00	0.43

**Fig. 5:**
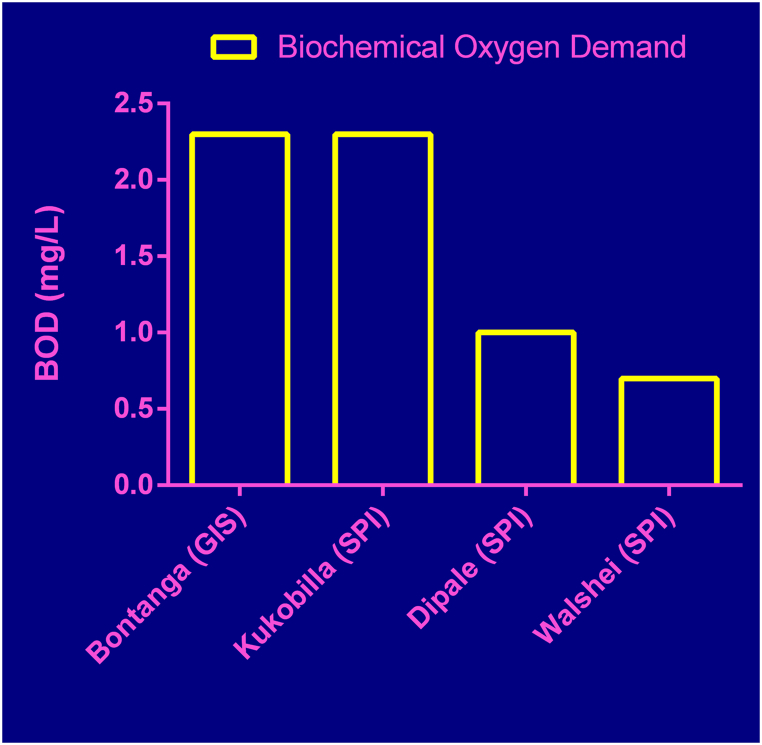
Biochemical Oxygen Demand (BOD) at GIS and SPI sites.

The BOD for GIS was 2.3 mg/l whilst those of the three selected small-private irrigation sites indicated 2.3 mg/l, 1.0 mg/l and 0.65 mg/l respectively for Kukobilla, Dipale and Walshei sites (Fig. 5). The three small private irrigation sites represent upstream, midstream and downstream reach respectively of the catchment under study along the White Volta River.

## 5 Discussion

### 5.1 Influence of irrigation scheme type on land productivity indicators

The area cultivated by the GIS in this study area was about 14 times the area cultivated by the SPI. However, output per unit land was higher under the SPI than the GIS. The higher output per unit cropped area under SPI could be attributed to the high proportion of land under vegetable production which has a higher value compared to grain crops which covers higher area of production under the GIS. The entire land in SPI was put under vegetable cultivation whilst under GIS only 26% of the land was used for vegetable cultivation and 74% under rice production. Vegetable crops are usually more profitable as they attract a good market price [24]. As shown in Table 3, paddy rice had the least average market price of 0.6 Ghana cedis per kg while pepper had an average price of 3.8 Ghana cedis per kg. Hence, the choice of crop played a significant role in improving the land productivity of SPI. Government irrigation schemes in Ghana were established to meet food self-sufficiency of the nation, so the primary crop cultivated in most government schemes is paddy rice. Owusu et al. [25] indicated that the 22 formal irrigation systems under Ghana irrigation development authority (GIDA) cultivate rice as their main crop. This phenomenon has stayed for several decades and has become the common practice and so farmers find it difficult to change and follow current market trends. This is not an obligation though by management of the government irrigation schemes however the difficulty in change is typical of farmers in Ghana. The other factor why farmers continue to cultivate rice even though it has a low price per kg compared to vegetables could be that most farmers in the government scheme are subsistence farmers whose primary aim is to obtain food for the family. Despite the higher price for vegetables, it is also important to note that vegetable production has been characterized by diseases and price fluctuation in the region and farmers risk when cultivating vegetables. It can therefore be said that private irrigators take risk than their colleagues in the government schemes. Furthermore, even though the government scheme also cultivated some high value crops, comparatively, the SPI still made higher yields in crops such as pepper compared to the GIS, further enhancing the productivity under SPI.

The results of this study are comparable to other studies in the sub-region. In a similar study conducted by Molden et al. [9] which includes two sub-Saharan African countries i.e. Burkina Faso and Niger, the output per unit cropped area ranged between 771 US$/ha and 3085 US$/ha. In the same study, output per unit command area for irrigation schemes in Burkina Faso and Niger ranged between 679 US$/ha and 2652 US$/ha. Comparing their study to the current study, the GIS had low output per unit cultivated area and fairly good output per unit command area while the private irrigators had higher in terms of output per unit command area. Similarly, Dejen et al. [26] conducted a study of three selected irrigation schemes in Ethiopia and reported that output per unit cropped area ranged between 1650 US$/ha and 2660 US$/ha whilst output per unit command area ranged between 2000 US$/ha and 6000 US$/ha.

### 5.2 Influence of irrigation scheme type on water productivity indicators

Water productivity indicators were also higher under SPI than in GIS. Under GIS there was very low output per unit irrigation supply and output per unit water supply compared with that of the SPI as shown in Fig. 4. The low outputs per unit water used were influenced by the high production of paddy rice in the government scheme which is a high-water consumption crop. Crop water and irrigation requirements of rice are very high due to the inclusion of percolation losses into the water requirement of rice. This increased the average water use per ha in the government scheme compared to that of the small private irrigators. Also, SGVP is influenced by factors such as price, crop yield and area under cultivation. The higher the yield and price of a crop, the higher the SGVP, and vice versa. Meanwhile, high SGVP leads to higher productivity, hence, the low price of rice in the market coupled with low yields led to lower SGVP and eventually lower water productivities at the GIS. Furthermore, irrigation water pumped per ha in the small private irrigation was less due to farmers adoption of crops with less water consumption. This choice of crop is probably because of high cost of fuel for running water pumps. These results are in line with similar studies in other areas of Africa. Molden et al. [9] indicated that output per unit irrigation supply for Burkina Faso and Niger ranged between 0.05 US$/m3 and 0.37 US$/m3 whilst output per unit water consumed ranged between 0.11 US$/m^3^ and 0.91 US$/m^3^. In Ethiopia, Dejen et al. [26] showed that output per unit irrigation supply ranged between 0.11 US$/m^3^ and 0.33 US$/m^3^ whilst output per unit water consumed ranged between 0.33 US$/m^3^ and 0.48 US$/m^3^.

### 5.3 Impact of irrigation scheme type on water quality

The quality of water used for irrigation was assessed to measure the environmental performance as poor water quality will result in pollution of the environment and may also pose risk to human health. According to Malano and Burton [10], Electrical Conductivity (EC) and Biochemical Oxygen Demand (BOD) of irrigation water can be used as parameters for assessing environmental performance of irrigation systems. The EC of water sources of both the government scheme and the small private irrigation sites were less than 100 μS/cm. This is a safe level of irrigation water quality [27]. The EC is a measure of salt concentration of irrigation water. The higher the EC, the higher the salt concentration. In this study the EC values obtained implied that there is no significant risk of salinity since the EC values were less than 100 μS/cm. The BOD value shows the amount of dissolved oxygen required by aerobic microbial organisms in the water body to break organic matter in the water at a specific temperature for a specified period. It is also a measure for surface water quality for irrigation, domestic, animal watering and aquatic life. A high BOD value means high concentration of organic matter in the water, which will lead to increased demand for dissolved oxygen for microbial decomposition which can cause general shortage of oxygen for the aquatic species. Also increased BOD could affect soil quality by reducing oxygen content in the soil which can cause yield decline in the long run. The BOD values were between 0.7 and 2.3 mg/l for both GIS and SPI which falls within WHO safe standards for water quality. According to the WHO, BOD values between the range of 3.0–5.0 mg/l is classified as moderately clean, which can be used for both drinking and irrigation purposes while water BOD greater than 5 mg/l indicates the water is undergoing pollution by a neighboring source [28]. Runoffs from agricultural fields could wash Nitrogen and Phosphorus into nearby water bodies, however the low BOD values in this study implied that anthropogenic activities such as agriculture has no much impact on the quality of water in the river. By inference, irrigation activities along the river banks have not impacted much on the river water quality. This trend may however change should agricultural activities intensify in the catchment.

## 6 Conclusion

The results of this study show that land and water productivities of small private irrigation (SPI) systems in the Nawuni catchment along the White Volta sub-basin were significantly higher compared to the government irrigation scheme (GIS). This could be attributed to the choice of cultivating relatively higher economically valued crops. Farmers under SPI are business-oriented as their cultivation targets the prevailing market demand whilst farmers under GIS are conservative and do not seem to consider market factors in their production decisions. In order to improve the productivities of the government irrigation schemes in Northern Ghana, high value crops cultivation should be encouraged, especially in the dry season, in order to achieve “value for money”. The state of small private irrigation at the time of this study did not show any significant environmental threat, likewise the GIS, hence the sector could be a major source of food and fibre, employment and livelihood for surrounding communities, however, sustainable water management practices should be encouraged in order to protect the water bodies from pollution in case of future intensification in the basin. For the sustainability of both SPI and GIS in the catchment, we recommend training programmes and bye-laws on sustainable water management.

## Author contribution statement

Abdul-Rauf Malimanga Alhassan and Andrew Manoba Limantol: Conceived and designed the experiments; Performed the experiments; Analyzed and interpreted the data; Wrote the paper. Isaac Larbi: Conceived and designed the experiments; Analyzed and interpreted the data; Wrote the paper. Rosemary Anderson Akolaa and Gilbert Ayine Akolgo: Analyzed and interpreted the data; Wrote the paper.

## Data availability statement

Data associated with this study has been deposited at Figshare. The doi is 10.6084/m9. figshare.21,960,077.

## Declaration of competing interest

The authors declare that they have no known competing financial interests or personal relationships that could have appeared to influence the work reported in this paper.
