# Supplementary material for: Analyses of crop water use and environmental performance of small private irrigation along the white Volta basin of Northern Ghana
Source: Heliyon. 2023 Aug 16;9(8):e19181. doi: 10.1016/j.heliyon.2023.e19181 (PMC10458339; doi:10.1016/j.heliyon.2023.e19181)
Supplement: Multimedia component 1 [file mmc1.docx]

**Supplementary Files**

**Table S1. Crop Water Requirements of Crops under GIS and SPI**

| **Crop** | **Crop Water Requirement (ETc) (mm)** | **Irrigation Requirement (mm)** |
| --- | --- | --- |
| Pepper | 535.40 | 474.90 |
| Tomato | 667.40 | 564.5 |
| Okra | 479.60 | 439.3 |
| Onion | 415.50 | 400.80 |
| Rice | 760.1 | 916.8 |
