# Supplementary figures and images for: Analyses of crop water use and environmental performance of small private irrigation along the white Volta basin of Northern Ghana
Source: Heliyon. 2023 Aug 16;9(8):e19181. doi: 10.1016/j.heliyon.2023.e19181 (PMC10458339; doi:10.1016/j.heliyon.2023.e19181)

**Table S2. Sample calculation of Crop Water Requirement of Pepper**


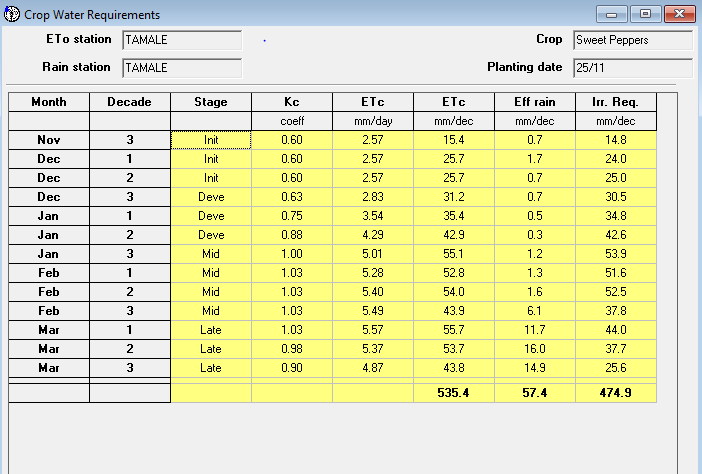

Supplement: Multimedia component 2 [file mmc2.docx]
