# Supplementary material for: Analyses of crop water use and environmental performance of small private irrigation along the white Volta basin of Northern Ghana
Source: Heliyon. 2023 Aug 16;9(8):e19181. doi: 10.1016/j.heliyon.2023.e19181 (PMC10458339; doi:10.1016/j.heliyon.2023.e19181)
Supplement: Multimedia component 3 [file mmc3.docx]

##### QUESTIONNAIRE FOR DATA COLLECTION OF IRRIGATION ALONG THE WHITE VOLTA OF NORTHERN GHANA

This study is conducted to assess the performances of small-private irrigation systems in comparison with government-led irrigation systems. Your participation in the study is considered voluntary and you reserve the right to terminate your participation at any point in time. Your confidentiality and anonymity are assured. The study is mainly for academic purpose. Thank you.

**DATE:**

##### DEMOGRAPHIC DATA OF RESPONDENT

Name:

Age:

Sex:

##### Location/ Land acquisition

Administrative District:

Type of Scheme:

Scheme/Site/Community name:

Number of Households:

How did you acquire the land to establish your farm?

- 1. From Government b. From Chief c. From fellow farmers d. None Did you pay for the acquisition? Yes. No.

Do you as farmers acquire permit before establishing farms within this site?

Yes No

If yes, from which agencies did you take permit?

a. MoFA b. VBA c. WRC d. EPA

##### Socio-Economic

What farming system are you practicing?

- 1. Cash Cropping b. Subsistence c. Mixed cash/subsistence

##### Water Source

Where do you get water for your irrigation?

- 1. Reservoir b. River c. Groundwater d. Conjunctive

##### Method/Amount of water abstraction and Irrigation

What is your method of water abstraction?

- 1. Pump b. Gravity c. Watering can/buckets d. Others

If you use a pump, what is the design flow rate of your pump?

How many times do you practice this irrigation in a year? a. Once b. Twice

What is the duration of each season in days? Season 1................Season 2......................

How many times do you irrigate your crops per week? ...............................................

What is the average number of hours of Irrigation per hectare?...........................................

Do you have discharge measurement facilities? Yes........ No.............

What is the method you use for field irrigation?

a. Furrow b. Basin c. Border d. Flood e. Sprinkler f. Drip

##### Total Area

| Command Area | Year 1 | | Year 2 | | Year 3 | |
| --- | --- | --- | --- | --- | --- | --- |
|  |  | |  | |  | |
| Irrigated Area | Year 1 | | Year 2 | | Year 3 | |
|  | Season A | Season B | Season A | Season B | Season A | Season B |
|  |  |  |  |  |  |  |
| Total number of farmers | Year 1 | | Year 2 | | Year 3 | |
|  |  | |  | |  | |
| Average Farm size |  | |  | |  | |
|  |  | |  | |  | |
| Total Number of Farmers | M | F | M | F | M | F |
|  |  |  |  |  |  |  |

1. **Crops grown, yield and price.**

| **Crop** | **Command Area (Ha)** | **Irrigated Area Ha**  **(Season 1 & 2)** | | **Average Yield (Bags/buc kets)** | **Weight of Bag/bucket** | **Production (kg)** | **Unit Price in GHc/kg** |
| --- | --- | --- | --- | --- | --- | --- | --- |
|  |  | **1** | **2** |  |  |  |  |
|  |  |  |  |  |  |  |  |
|  |  |  |  |  |  |  |  |
|  |  |  |  |  |  |  |  |
|  |  |  |  |  |  |  |  |
|  |  |  |  |  |  |  |  |
|  |  |  |  |  |  |  |  |

1. **Cropping Calendar**

| **Crop** | **Planting date (Range)** | **Harvesting date (Range)** | **Total Growing days** |
| --- | --- | --- | --- |
|  |  |  |  |
|  |  |  |  |
|  |  |  |  |
|  |  |  |  |
|  |  |  |  |

## Measurements Sheet for Canal Discharge

### Equipment

**Float Method of Discharge Measurements**

### Date:

30 m long measuring tape Stop watch

Floating Object (Orange) Wooden Stakes **Procedure**

Choose a uniform section along the canal. Choose the section a bit far from check gates or division boxes.

Choose a length and label it as L and place stakes from point 1 to point 2.

Use a stop watch to observe the time traveled by the floating object from point 1 to point 2. Repeat the above steps and record the data for several observations

Measure the top width and bottom width of the canal as well as the water level at various sections of the canal.

Calculate the average cross section using the formula below

𝐴 = 𝑎 + 𝑏 𝑥 ℎ 2

Compute the average velocity of the flow using the relation below

𝑉_ƒ_ = 𝑉_𝑚_ 𝑥 ƒ

Where

*V_f_ is the actual velocity of the flow*

*V_m_ is the surface velocity of the flow*

*f* is the correction factor which ranges between 0.65 to 0.80

1. Right Bank Canal
2. Length of Canal Section, L=
3. Float travel time

| No. | Time (seconds) | Remarks |
| --- | --- | --- |
| 1 |  |  |
| 2 |  |  |
| 3 |  |  |
| 4 |  |  |
| 5 |  |  |
|  |  |  |
|  |  |  |

1. Left Bank Canal
2. Length of Canal Section, L=
3. Float travel time

| No. | Time (seconds) | Remarks |
| --- | --- | --- |
| 1 |  |  |
| 2 |  |  |
| 3 |  |  |
| 4 |  |  |
| 5 |  |  |
|  |  |  |
|  |  |  |

Canal Cross-Section-Right Bank

|  | Cross-Section 1 | Cross-Section 2 | Cross-Section 3 |
| --- | --- | --- | --- |
| a |  |  |  |
| b |  |  |  |
| h |  |  |  |

Canal Cross-Section-Right Bank

|  | Cross-Section 1 | Cross-Section 2 | Cross-Section 3 |
| --- | --- | --- | --- |
| a |  |  |  |
| b |  |  |  |
| h |  |  |  |
